# Safety Evaluation and Probabilistic Health Risk Assessment of Cow Milk Produced in Northern Italy According to Dioxins and PCBs Contamination Levels

**DOI:** 10.3390/foods12091869

**Published:** 2023-04-30

**Authors:** Maria Olga Varrà, Valentina Lorenzi, Emanuela Zanardi, Simonetta Menotta, Giorgio Fedrizzi, Barbara Angelone, Mara Gasparini, Francesca Fusi, Stefano Foschini, Anna Padovani, Sergio Ghidini

**Affiliations:** 1 Department of Food and Drug, University of Parma, 43124 Parma, Italy; mariaolga.varra@unipr.it (M.O.V.); emanuela.zanardi@unipr.it (E.Z.); sergio.ghidini@unipr.it (S.G.); 2 Istituto Zooprofilattico Sperimentale della Lombardia e dell’Emilia Romagna “Bruno Ubertini”, 25124 Brescia, Italy; simonetta.menotta@izsler.it (S.M.); barbara.angelone@izsler.it (B.A.); mara.gasparini@izsler.it (M.G.); francesca.fusi@izsler.it (F.F.); 3 Chemical Department, Istituto Zooprofilattico Sperimentale della Lombardia e dell’Emilia-Romagna “Bruno Ubertini”, 40127 Bologna, Italy; giorgio.fedrizzi@izsler.it; 4 Unità Organizzativa Veterinaria, Direzione Generale Welfare Regione Lombardia, 20124 Milano, Italy; stefano.foschini@regione.lombardia.it; 5 Area Sanità Veterinaria e Igiene degli Alimenti, Settore Prevenzione Collettiva e Sanità Pubblica, Direzione Generale Cura della Persona, Salute e Welfare, Regione Emilia Romagna, 40127 Bologna, Italy; anna.padovani@regione.emilia-romagna.it

**Keywords:** dioxins, risk assessment, food contaminants, chemical risk, dairy, bovine, Monte Carlo simulations

## Abstract

Contamination levels of dioxins and polychlorinated biphenyls (PCBs) were monitored over 2018–2021 in 214 bovine milk samples from farms located in two regions in northern Italy (Lombardy and Emilia-Romagna). The average concentrations of the sum of dioxins and dioxin-like PCBs (0.78 ± 0.55 pg TEQ/g fat) and six non-dioxin-like PCBs (6.55 ± 2.24 ng/g fat) were largely below the maximum, and action limits established at European level, confirming a decreasing trend observed both locally and across Europe in recent years. The impact of contamination levels on chronic dietary exposure of the Italian population to dioxins and PCBs was found to be highly variable based on the type of cow milk (skimmed, semi-skimmed, or whole-fat milk) and the population age group considered. Indeed, a first-tier screening of the potential exposure via determinist methods allowed for the identification of the youngest population as the group with the worst risk profile. The refinement of exposure assessment via Monte Carlo probabilistic methods suggested that, at the less pessimistic middle-bound simulation scenario, infants, toddlers, and children consuming whole cow milk may be exposed to dioxins and PCBs levels above the toxicological reference values with a probability of 76, 56, and 22%, respectively.

## 1. Introduction

Polychlorinated dibenzo-p-dioxins and furans (PCDD/Fs, also known as dioxins) and polychlorinated biphenyls (PCBs) are persistent organic pollutants widely distributed in the environment. Even though they include a wide range of chemical compounds, they all have low degradation potentials and high lipophilic character. Furthermore, PCDD/Fs and PCBs have similar chronic toxic effects on animals and humans, potentially causing developmental and reproductive disorders, endocrine and immune system impairment, teratogenesis, and tumorigenesis [1,2,3,4].

The most toxic congeners are 2,3,7,8-tetrachlorodibenzo-p-dioxin (TCDD) and 1,2,3,7,8-pentachlorodibenzo-p-dioxin (PCDD) [5], with an estimated half-life in the human body of approximately 10 years after the initial exposure [2,6].

Due to their high resistance to metabolic degradation and lipophilic behavior, these compounds easily accumulate in food and feed chains [7]. Milk and derived products were reported as the first dietary contributors to the total body burden of dioxins and PCBs for infants, toddlers, and children (28–50%), but also impactful for older European population groups (7–25%) [3].

The presence of dioxins and PCBs in the fat fraction of cow milk in considerable amounts is the consequence of the active excretion of these compounds by the animals, which use this mechanism as an elimination route [8]. Sources of dioxins and PCBs for cattle are pasture grass for free-range animals and mainly silage feedstuffs and feed additives for intensively farmed ones [9,10,11]. More specifically, certain types of feed such as citrus pulp, clay minerals, breadcrumbs, potato peelings, corn, sugar beet pulp, and certain fats and oils have been found to contain high levels of PCDD/Fs and PCBs [12,13,14,15,16]. Dried materials such as corn, sugar beet pulp, grass, alfalfa, and breadcrumbs are particularly susceptible to contamination due to the possible formation of PCDD/Fs if they are not dried properly [17]. In turn, the source of feed contamination may be ascribed to emissions from both industrial and non-industrial sources (e.g., coal burning in furnaces, domestic waste burning, and car exhausts) [18]. In this setting, it has been widely demonstrated that concentrations in animal-derived products are frequently higher than in the feed material carrying them [18,19,20]. Many factors related to the physiology, biology, and management of animals, including age, grazing habits, breeding characteristics, lactation stage, body weight, body fat content, and milkfat production, may contribute to the presence of dioxins and PCBs in animal tissues and milk [21,22,23,24]. However, there is not enough conclusive evidence to determine the specific influence of these factors since most studies do not focus on investigating them [24]. Conversely, greater emphasis has been placed on how the contamination of milk with dioxins and PCBs can vary depending on geographic location and season. Even small, periodic changes in contamination levels have been observed, but these were mostly attributed to fluctuations in the concentrations of contaminants in the feed consumed by the cattle, as well as differences in pollutant exposure between indoor and outdoor environments during different seasons [25,26,27].

The milk and dairy sectors are an important part of the Italian economy, with an annual production of thirteen million tons of bovine milk, mainly intended for fresh consumption or cheese making [28]. Specifically, approximately 20,000 cow farms are spread across the northern Italian regions of Lombardy and Emilia-Romagna, which alone account for more than 60% of national milk production [29]. The Italian population, therefore, has a high consumption of milk and dairy products, and exposure scenarios to dioxins and PCBs through the consumption of these food items may be of particular concern compared to other European citizens.

To reduce the concentrations of dioxins and PCBs along the whole food chain, risk management measures have been implemented in the European Union. First, maximum limits (MLs) and action limits (ALs) were established to prevent contaminated foodstuffs and feedstuffs from being placed on the market and to improve the identification and removal of pollution sources. Although, in the last few years, these measures have led to a general reduction in human assimilation of dioxins through the diet [3], it should be noted that MLs have been set according to the so-called ‘strict but feasible’ principle, i.e., they have been established at the 90th percentile of their frequency distribution in each specific food item without following toxicologically driven criteria [3,30]. Moreover, despite the reduction in dietary exposure to dioxins and PCBs, the European Food Safety Authority (EFSA) has recently estimated that the tolerable weekly intake (TWI) of these contaminants continues to be exceeded for most of the European population and, therefore, the Authority has decided to drastically reduce the TWI of the sum of PCDD/Fs and dioxin-like (dl)-PCB to 2 pg toxic equivalent (TEQ)/kg bw/week [31]. As a result, the European Commission lowered the MLs for several food commodities in 2022. For milk and dairy, MLs of 2 and 4 pg TEQ/g fat of PCDD/Fs and PCDD/Fs + dl-PCB, respectively, and of 40 ng/g fat for non-dioxin-like (ndl)-PCBs are currently in force [32].

In light of new TWI and MLs limits, as well as changes in the dietary habits of the Italian population, it is therefore important to pursue risk management strategies through the ongoing monitoring of dioxin and PCB contamination levels in food and feed to measure the public health impact of dietary exposure to these contaminants and to identify those segments of the population that are at increased risk of exposure and potentially long-term toxic effects. In this context, probabilistic risk assessment might provide more confident and realistic representations of the actual risk levels to which consumers are exposed, accounting for the variability and uncertainty of exposure estimates.

In this work, the results of the 2018–2021 monitoring plan on dioxin and PCB residues carried out in the northern Italian regions of Lombardy and Emilia Romagna are presented, with the aim of evaluating the occurrence, spatial distributions, and temporal variations of these contaminants in bovine milk produced in this territory. A second-level objective was to perform an exposure assessment of dioxins and PCBs intake through the consumption of milk with different fat contents by different population age groups in Italy and verify to what extent the consumption of milk contributes to the toxicological reference values using both deterministic and probabilistic (Monte Carlo method) approaches, providing a realistic scenario of the possible risks.

## 2. Materials and Methods

### 2.1. Milk Samples

**Figure 1:**
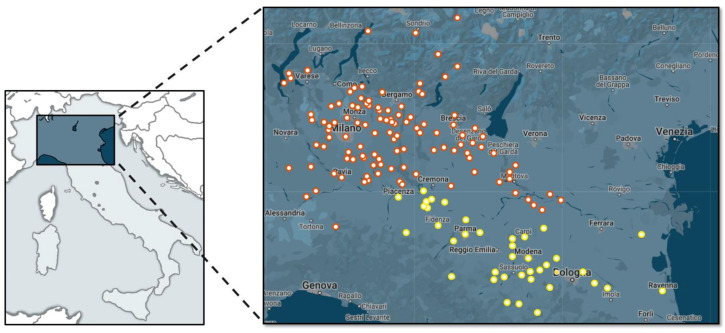
Geographical distribution of bovine farms of the northern Italian regions (Lombardy, red dots; Emilia-Romagna, yellow dots) from which milk samples (N = 214) were collected within 2018–2021.

Milk samples were collected from 2018 to 2021 under the framework of the Italian residue monitoring plan (RMP). In those years, the Italian RMP was structured according to Council Directive 96/23/EC [33] and Commission Decision 97/747/EC [34], as well as Regulation (EU) 625/2017/EU [35]. A total of 214 bovine milk samples were obtained from 187 dairy farms in the Lombardy and Emilia-Romagna regions of Northern Italy (Figure 1). Milk samples were collected from bulk farm tanks by the official competent authorities according to Regulation (EU) 644/2017/EU [36]. Specifically, bulk milk was thoroughly mixed in order to ensure and homogeneous distribution of the contaminants, and an aggregate sample of 1 L was obtained from each farm. Once collected, the samples were stored at +4 °C and delivered within 24 h to the official local laboratory of the Istituto Zooprofilattico Sperimentale della Lombardia e dell’Emilia-Romagna, certified under ISO/IEC 17025:2017 and accredited for quantitative determination of PCDD/Fs (2,3,7,8-tetracholodibezodioxin (TCDD), 1,2,3,7,8-pentachlorodibenzodioxin (PeCDD), 1,2,3,4,7,8-hexachlorodibenzodioxin (HxCDD), 1,2,3,6,7,8-HxCDD, 1,2,3,7,8,9-HxCDD, 1,2,3,4,6,7,8-heptaclorodibenzodioxin (HpCDD), 1,2,3,4,6,7,8,9-octochlorodibenzodioxin (OCDD), 2,3,7,8-tetracholodibezofuran (TCDF), 2,3,4,7,8-pentacholodibezofuran (PeCDF), 1,2,3,7,8-PeCDF, 2,3,4,6,7,8-hexacholodibezofuran HxCDF, 1,2,3,7,8,9-HxCDF, 1,2,3,6,7,8-HxCDF, 1,2,3,4,7,8-HxCDF, 1,2,3,4,7,8,9-heptacholodibezofuran (HpCDF), 1,2,3,4,6,7,8-HpCDF, 1,2,3,4,6,7,8,9-octacholodibezofuran (OCDF), dl-PCBs (PCB 77, 81, 105, 114, 118, 123, 126, 156, 157, 167, 169, 189) and ndl-PCBs (PCB 28, 52, 101, 138, 153, 180), using high-resolution gas chromatography coupled with high-resolution mass spectrometry (HRGC-HRMS).

### 2.2. Reagents and Standards

Ethyl-acetate, toluene, and n-hexane were purchased from Carlo Erba Reagents (Milan, Italy); nonane from Promochem (LGC Standards, Teddington, UK) and dichloromethane from Romil Ltd. (Cambridge, UK). All solvents were picograde.

Pre-packed multi-layer silica, alumina, and carbon columns were produced by FMS (Fluid Management System, Billerica, MA, USA). The ^13^C-labeled recovery, clean-up, and standard injection solutions were provided by CIL (Cambridge Isotope Laboratories, Andover, MA, USA). For PCDD/Fs, EDF-9999 Method 1613 calibration solutions (CS1-CS5) were used. For PCB calibration, an in-house curve was prepared using PCB MIX-75 (Dr. Ehrenstorfer, Augsburg, Germany) and ^13^C-labeled solutions EC-4995 and EC-4978 (Cambridge Isotope Laboratories, Andover, MA, USA).

All solvents and reagents used for the analyses were tested to ensure the absence of contaminants at the levels of interest (i.e., below one-fifth of the limit of quantification (LOQ) for PCDD/Fs and below one-tenth of the LOQ for dl-PCB and ndl-PCB).

### 2.3. Analytical Method

The methods used for the determination of PCDD/Fs and PCBs in milk samples were previously described by Lorenzi et al. [20,37]. Briefly, milk samples were homogenized and freeze-dried (Freeze Dryer Martin Christ Gefriertrocknungsanlagen, Osterode am Harz, Germany). Following further homogenization, a portion of each sample was tested with the Soxhlet method to determine the lipid content, while 8–10 g of the samples were mixed with diatomaceous earth and spiked with the ^13^C-labeled internal standard EDF-8999 (Cambridge Isotope Laboratories, Andover, MA, USA) and the ^13^C-labeled internal standard EC-4995 (Cambridge Isotope Laboratories, Andover, MA, USA). An accelerated solvent extractor (ASE^®^ 300, Sunnyvale, Dionex, CA, USA) was used for fat extraction with toluene (2 cycles of 20 min at +135 °C and 1500 psi). The solvent was filtered using anhydrous sodium sulfate and evaporated at +50 °C with a rotatory evaporator. The lipid extracts were dried overnight at 70 °C ± 5 °C. Fat content was determined gravimetrically and compared to the value obtained from the Soxhlet method to confirm ASE extraction efficiency.

Lipid extracts were solubilized in 5 mL of hexane/dichloromethane solution (1:1, *v/v*), spiked with the standard clean-up solution EC-4978 (Cambridge Isotope Laboratories, Andover, MA, USA), containing three ^13^C-labeled PCB congeners, and diluted with 20 mL of hexane. The diluted extracts were purified using pre-packed silica columns, acidified with sulfuric acid, and eluted with n-hexane. The purification fractions were concentrated to 0.5 mL in the TurboVap evaporator (Zymark, Hopkinton, MA, USA) and loaded into the Power-Prep^™^ system (Fluid Management System, Watertown, MA, USA) equipped with silica, alumina, and carbon columns. Toluene (50 mL) was used to elute PCDD/Fs from the carbon column, while n-hexane and a mixture of hexane/dichloromethane solution (9:1, *v/v*) were used for PCB elution from the alumina column. The final extracts were dried using the TurboVap evaporator and a vacuum concentrator (Genevac, Ipswich, UK).

The PCDD/F fraction was dissolved in 10 μL of 1:100 ED-2521 injection solution (Cambridge Isotope Laboratories, Andover, MA, USA), and the PCB fraction was dissolved in 20 μL of 1:50 EC-4979 injection solution (Cambridge Isotope Laboratories, Andover, MA, USA). HRGC-HRMS analysis was carried out using two TRACE GC ULTRA gas chromatography coupled with a DFS (double focusing system) high-resolution magnetic scan system (Thermo Fisher Scientific, Waltham, MA, USA) or with AutoSpec high-resolution mass spectrometer (Micromass/Waters, Manchester, UK) [24]. A DB5 MS capillary column (60 m × 0.25 mm, 0.25 µm, Thermo Fisher Scientific, Waltham, MA, USA) was used for PCDD/F separation, and a TR-PCB 8MS capillary column (50 m × 0.25 mm, 0.25 µm, Thermo Fisher Scientific, Waltham, MA, USA) for PCB separation. The mass spectrometer operated in selected ion monitoring mode with a mass resolution of over 10,000.

Results were expressed in pg/g fat for PCDD/Fs and dl-PCBs and ng/g fat for ndl-PCBs. Toxic equivalent values (TEQ) were calculated using the 2005 World Health Organization Toxic Equivalency Factors (WHO-TEFs) [5].

### 2.4. Quality Control

The laboratory participated in different international proficiency tests and inter-laboratory studies, obtaining satisfactory results.

To ensure the absence of cross-contamination and interferences, blank samples were processed with each batch of five samples. For every ten samples, a fortified sample was processed to ensure analyte recovery. The stability of response factors for all congeners was checked daily using calibration verification standard solutions for PCDD/Fs and PCBs and assessing the acceptability of deviations from the calibration curves [38,39]. The recovery values of the internal standard congeners were determined for each sample.

Duplicate analyses were performed on each sample, and the compliance of the absolute difference between the two measurements with the repeatability limits, obtained during the validation study, was used to estimate the overall repeatability of the method.

LOQ and recovery values are reported in Supplementary Table S1.

### 2.5. Summary and Statistics

Contamination levels of dioxins and dl-PCBs were reported both as actual mass fraction values (pg/g fat) and toxic equivalency values (pg TEQ/g fat), while those of ndl-PCBs as actual mass fraction values (ng/g fat).

Non-detected values, i.e., values below the LOQ, were substituted with the LOQ (upper bound concentrations, UB), ½ LOQ (middle bound concentrations, MB), or 0 (lower bound concentrations, LB). Univariate descriptive statistics were applied to the UB-data matrix to identify potential differences among the tested contaminants. The Student’s T test was employed to evaluate differences between the two Italian regions from which samples were collected (Lombardy and Emilia-Romagna). The Analysis of Variance (ANOVA) followed by Tukey’s post hoc test was used to identify the provinces of each Italian region characterized by the highest concentrations of contaminants and to explore significant differences over the 2018–2021 sampling years. A Spearman’s correlation analysis was performed to investigate potential links between the fat content and dioxin concentrations in milk samples. PCDD/F + dl-PCB concentrations (pg/g fat) of each sample were multiplied by the relative fat content (g fat/100 g) to calculate the overall PCDD/Fs + dl-PCBs in 100 g of milk, and then these results were correlated with the respective lipid content. Correlation coefficients (*r*) higher or lower than 0.6 were considered indicative of the existence of a positive or negative correlation, respectively. The statistical significance was set at *p* ≤ 0.05 for all tests.

Statistical analysis was performed using OriginPro 2021 software (v. 9.8.0.200, OriginLab, Northampton, MA, USA).

### 2.6. Exposure Assessment and Risk Characterisation

#### 2.6.1. Screening via Deterministic Approach

The estimated weekly intakes (EWIs) of PCDD/Fs + dl-PCBs through milk consumption were calculated using the measured PCDD/Fs + dl-PCBs mean (UB) concentrations, milkfat concentrations, and mean or 95th percentile chronic dietary consumption data of cow milk by the Italian population.

Consumption data of cow milk (consumers only) were retrieved from a more recent Italian survey on food consumption SCAI IV included within the EFSA Comprehensive Food Consumption Database [40,41,42]. Infants (up to 12 months old), toddlers (13–36 months old), children (37 months–9 years old), adolescents (10–17 years old), adults (18–65 years old), and the elderly (>65 years old) were the six Italian population age groups considered.

The cow milk collected in this study was supposed to be destined for the production and commercialization of milk with different fat percentages. Therefore, dietary consumption data of skimmed, semi-skimmed, and whole cow milk were retrieved from Level 6 of the FoodEx2 hierarchical classification system included in the EFSA Database and used to simulate three different chronic exposure scenarios per population group. For the calculation of EWIs, skimmed, semi-skimmed, and whole cow milk were supposed to contain 0.3, 1.8, and 3.5 g lipids/100 g, respectively, by using Equation (1):

EWI (pg TEQ/kg bw/week) = C × M × F ÷ 100,(1)

where C is the mean concentration of PCDD/Fs + dl-PCBs (pg TEQ/g fat); M is the mean or 95th percentile chronic weekly consumption of skimmed, semi-skimmed, or whole cow milk (g/kg bw/week); F is the fat content of skimmed, semi-skimmed, or whole cow milk samples (g fat/100 g).

Finally, the risk was characterized by comparing the calculated EWI with the TWI of PCDD/Fs + dl-PCBs (equal to 2 pg/kg bw/week) by using Equation (2):

TWI (%) = (EWI ÷TWI) × 100.(2)

#### 2.6.2. Refinement via Probabilistic Methods (Monte Carlo Simulations)

The Monte Carlo (MC) probabilistic method was applied to simulate the distribution probabilities of EWI values and the related %TWI only for those deterministic scenarios indicating potential causes of concern, as suggested by EFSA [43]. Rather than using single point estimates, the distribution frequency of both the PCDD/Fs + dl-PCB and chronic consumption data of cow milk (i.e., assumptions) were considered. In this way, information concerning uncertainty, variability, and probability in health risk evaluation was obtained.

First, the best-fit probability distributions for the PCDD/Fs + dl-PCB contamination levels were tested using the modified Kolmogorov–Smirnov goodness-of-fit test (*p* > 0.05). In this case, the LB, MB, and UB concentrations were individually used to account for uncertainty embedded within the data. Based on the results, the LB, MB, and UB concentrations were fitted to the lognormal distribution. No distribution frequencies were set for milkfat, and fixed values at 1.8 and 3.5 g/100 g for semi-skimmed and whole cow milk were used. The overall distribution frequency of dietary consumption data for milk (expressed as g/kg bw/week) was supposed to be lognormal based on the literature data [44,45].

After that, MCSs were run randomly and repeatedly using 50,000 iterations for each population subgroup using Equations (1) and (2) so as to forecast a set of theoretically stable distributions of PCDD/F + dl-PCB EWIs and relative contributions to TWI (%), as well as their probabilities at different risk levels (10–90th percentiles).

All the analyses were performed using the Crystal Ball software (v. 11.1.2.4, Oracle©, Austin, TX, USA).

## 3. Results and Discussion

### 3.1. Spatial and Temporal Distribution of Dioxins and PCBs in Milk

The average concentrations of dioxins and PCBs measured in the 214 cow milk samples are reported in Table 1, where they were statistically summarized by the Italian region (i.e., Lombardy vs. Emilia-Romagna) and the year of sampling (i.e., 2018 vs. 2019 vs. 2020 vs. 2021). Individual concentrations of each of the 35 measured PCDD/F, dl-PCB, and ndl-PCB congeners are provided in Supplementary Tables S2 and S3. Regardless of both the region and the year, the average concentrations of PCDD/Fs, dl-PCBs, PCDD/Fs + dl-PCBs, and ndl-PCBs were found to be 0.21 pg TEQ/g fat, 0.54 pg TEQ/g fat, 0.75 pg TEQ/g fat, and 0.64 ng/g fat, respectively (Table 1).

No statistically significant differences (*p* > 0.05) concerning concentrations in all the tested contaminants were found according to the four years of sampling. On the contrary, milk samples from Emilia-Romagna were found to be significantly less contaminated (*p* ≤ 0.05) by both dioxins and PCBs than those collected from the Lombardy region. Indeed, as can be observed in Table 1, milk samples from Emilia-Romagna presented a 20% lower concentration of PCDD/Fs (0.19 vs. 0.23 pg TEQ/g fat), a 40% lower concentration of dl-PCBs (0.41 vs. 0.66 pg TEQ/g fat), 30% lower concentrations of PCDD/Fs + dl-PCBs (0.60 vs. 0.88 pg TEQ/g fat), and 7% lower concentrations of ndl-PCBs (6.25 vs. 6.71 ng/g fat).

A deeper analysis of data was conducted to investigate whether the increased contamination of milk with PCDD/Fs + dl-PCBs was proportional to the lipid content of milk. A Spearman correlation analysis between the sample contamination levels and the lipid content was performed. Interestingly, the analysis showed a positive correlation between contamination levels and fat content only in milk samples from Emilia-Romagna (*r* = 0.63, *p* ≤ 0.05), while no significant correlations were found in samples from Lombardy (*r* = 0.11, *p* > 0.05). These findings suggest that Emilia-Romagna may have a relatively consistent and stable level of background contamination to the extent that the fat content of milk from this region could be somewhat indicative of the degree of dioxin contamination. In contrast, milk samples from Lombardy may be affected by background and point contamination, which could explain why some samples with low-fat content showed high contamination levels while some samples with high-fat content showed low contamination levels. This situation may be attributed to the higher level of industrialization in Lombardy, the existence of contaminated sites that produced PCBs in the past, and the proximity of industrial facilities to dairy farms [46].

**Figure 2:**
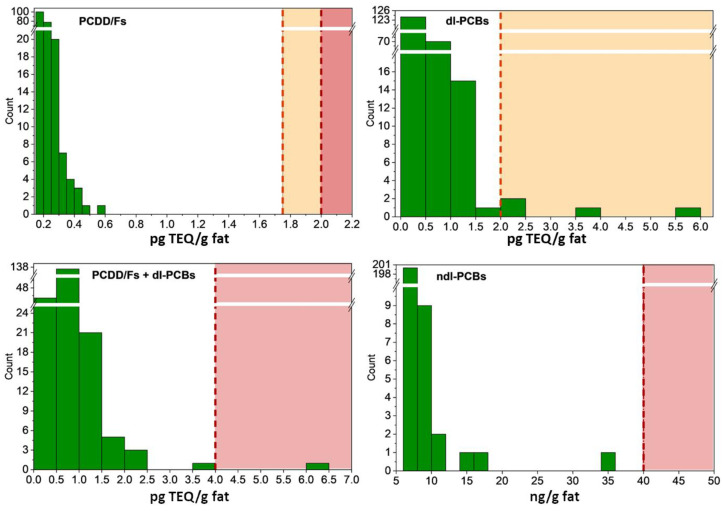
Frequency distribution of milk samples (N = 214) by contamination levels of PCDD/Fs (pg TEQ/g fat), dl-PCBs (pg TEQ/g fat), PCDD/Fs + dl-PCBs (pg TEQ/g fat), and ndl-PCBs (ng/g fat). Vertical dashed yellow and red lines mark the exceedance of action levels and maximum levels according to European Union requirements.

All milk samples were compliant with the European MLs of PCDD/Fs of 2 pg TEQ/g fat and ALs of 1.75 pg TEQ/g fat, as well as with the European MLs of ndl-PCBs of 40 ng/g fat. Nonetheless, one sample from Lombardy exceeded the MLs of 4 pg TEQ/g fat established for PCDD/Fs + dl-PCBs since contaminated by 6.28 pg TEQ/g fat, while the other four samples from Lombardy were above the ALs of 2 pg TEQ/g fat established for dl-PCBs (Figure 2).

When comparing cow milk contamination levels found in the present work with those reported elsewhere in the world, a quite variable scenario emerged. For example, in eight Brazilian states, cow milk samples were found to have a PCDD/Fs contamination level of 1.36 pg TEQ/g fat, which is approximately seven times higher than the concentration found in this study [10]. The authors attributed the higher contamination levels to the increased proximity of farms to urban centers and the degree of industrialization in the area [10]. In contrast, cow milk samples collected from seven different regions of Chile showed concentrations of PCDD/Fs + dl-PCBs up to 0.45 pg TEQ/g fat, two times lower than those found in this study [47]. The authors confirmed that higher contamination levels were observed in milk samples collected from more populated and industrialized areas [47].

When comparing data to those reported by Asian countries, it was observed that the PCDD/F levels of milk samples from traditional markets around Taiwan tended to be three-four times higher (0.59–0.89 pg TEQ/g fat) than those found in this study [48,49] but no significant differences in terms of dl-PCBs were identified [49]. Significantly lower PCDD/F + dl-PCB concentrations were recorded in milk samples from Malaysia (0.16 pg TEQ/g fat) [50], while concentrations of 0.13 ± 0.10 were reported in Chinese milk samples by Zhang et al. in 2015. However, since that value was expressed on a fresh weight rather than a lipid basis, it should be considered higher than those found in the present work [51].

In Europe, the EFSA reported a mean concentration of PCDD/Fs + dl-PCBs of 1.91 pg TEQ/g fat in milk and dairy products in 2012, which is higher compared to the contamination levels found in this study but includes contributions from milk-derived products (that usually have higher contamination levels than raw milk due to a larger amount of fat) [3]. In the same period, Marin et al. reported an average concentration of PCDD/Fs + dl-PCBs in milk and dairy products from Spain ranging from 0.25 pg TEQ/g fat to 2.13 pg TEQ/g fat [52].

Very high PCDD/F + dl-PCB contamination levels (up to 2–3 pg TEQ/g fat) were also reported in cow milk samples from Italy [7,53], as well as contamination levels up to 5.36 pg TEQ/g fat in buffalo milk from a contaminated site in southern Italy [54]. However, it is important to note that these data were calculated using the old toxic equivalency factors (TEFs) established in 1998 rather than those established in 2005 and currently employed [5]. Therefore, comparisons should be conducted with caution, especially since these surveys were conducted approximately 15 years ago and may not reflect the reduction trend observed in Europe in recent years.

Dioxin and PCB contamination levels in cow milk from Lombardy and Emilia-Romagna collected in this work (2018–2021) and the former monitoring plan (2012–2014) [37] were further compared. As can be observed from Table 2, concentrations of all the analyzed contaminants were significantly lower in 2018–2021 than in the 2012–2014 milk samples, with the highest reduction in dl-PCBs and PCDD/Fs + dl-PCBs actual mass values (pg/g fat or ng/g fat) recorded in milk samples from the Lombardy region (–70% in Lombardy vs. –53% in Emilia-Romagna). Similarly, compared to the 2012–2014 concentrations, the 2018–2021 ndl-PCB values fell to 6.71 ng/g fat (–37%) in Lombardy and to 6.25 ng/g fat (–19%) in Emilia Romagna. The highest PCDD/Fs decrease per year was instead observed in samples from Emilia Romagna (–44%) (Table 2).

The total TEQ values (pg TEQ/g fat) of both PCDD/Fs and dl-PCBs in the 2018–2021 milk samples from the two regions under study declined in direct proportion but less strongly than the actual mass values. Indeed, compared to the 2012–2014 outcomes, Lombardy and Emilia-Romagna saw PCB levels drop by 42% and 37%, respectively (Table 2), suggesting that this drop was mostly due to less toxic PCDD/F and dl-PCB congeners (i.e., congeners having lower TEF values and, hence, a lower impact on total TEQ values).

#### Variation of Dioxins and PCB Congener Patterns

Since each dioxin and PCB congener bears the imprint of the contamination source (geographical location and industrial activities) and is characterized by a different toxicological effect, monitoring dioxin and PCB congener patterns in food is crucial to understand better their presence in the environment, contamination sources, and the potential risks to human health. Numerous studies have suggested that the chemical species of dioxins and PCBs may vary significantly depending on the foods being considered [3]. Regarding cow milk, dioxin, and PCB chemical species might differ based on a number of variables, including the feeding regimen, age, and breed of the animal, as well as the ambient conditions in which the cattle are grown, the sampling, and the analytical techniques employed for the quantification [55].

**Figure 3:**
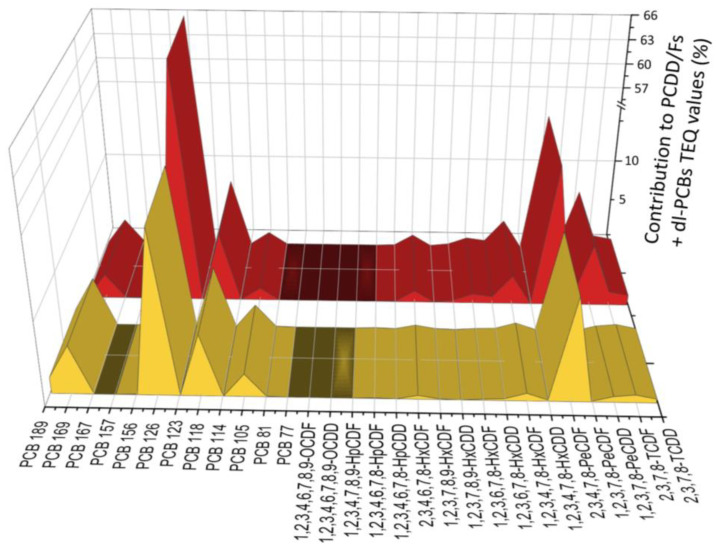
Relative percentage contribution of the 29 PCDD/F and dl-PCB congeners to the total PCDD/F + dl-PCB TEQ values (pg TEQ/g fat). Lombardy in red, Emilia Romagna in yellow.

**Figure 4:**
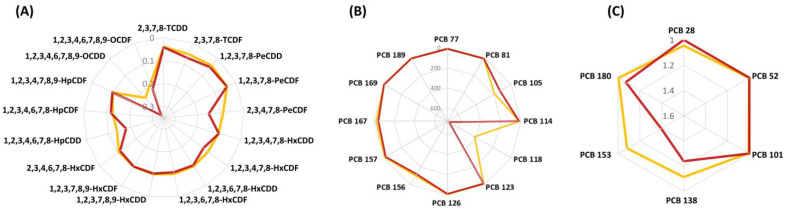
Radar chart showing congener profiles of PCDD/Fs (pg/g fat, (**A**)), dl-PCBs (pg/g fat, (**B**)), and ndl-PCBs (ng/g fat, (**C**)) in milk samples. Lombardy in red, Emilia Romagna in yellow.

To provide complementary information, dioxin and PCB congener profiles in cow milk from the two Italian areas were examined for their variation based on the sample sites and investigated by plotting both the relative percentage contribution of each congener to the total PCDD/Fs + dl-PCBs TEQ values (Figure 3) and the absolute mass fraction concentrations (pg/g fat) (Figure 4).

As seen in Figure 3, PCB 126 was found to be responsible for 66% and 57% of the overall PCDD/Fs + dl-PCBs TEQ values in milk samples from Lombardy and Emilia-Romagna, respectively. These results contrast with those of Barone et al., according to whom cow milk showed a predominance of PCDD/Fs [56], but support earlier findings from our team, who found that PCB 126 was the predominant dl-PCB congener in milk samples in northern Italy [37,57]. Additionally, previous studies from other world regions identified PCB 126 and the other non-ortho PCBs as the congeners having the most impact on the total TEQ values in milk [47,58,59,60,61].

The 2,3,4,7,8-PeCDF was found to be the chemical species with the second largest contribution (13% in samples from Lombardy and 9% in samples from Emilia-Romagna). On the other hand, two mono-ortho species, PCB 189 and PCB 105, showed a greater contribution to total TEQ values in milk samples from Emilia-Romagna (Figure 3).

When analyzing PCDD/F patterns expressed on mass fraction concentrations (pg/g fat), particularly evident differences of 2,3,4,7,8-PeCDF, 1,2,3,4,7,8-HxCDF, 1,2,3,4,6,7,8-HpCDD, and 1,2,3,4,6,7,8,9-OCDD were noted, whose levels were greater in milk from Lombardy (Figure 4A). By plotting contributions to TEQ values, the differences of the latter species were not evident since they were obscured by other congeners dominating the pattern.

PCDDs have typically been linked to agricultural chemicals such as chlorophenols, whereas more mixed PCDD/F profiles have been associated with both waste incineration and the usage of chlorinated agricultural pesticides [55]. Nonetheless, it can be challenging to accurately connect the PCDD and PCDF congener profiles of a specific food or feed to its sources because pollution sources might be numerous and varied.

As highlighted in Figure 3, milk samples from Lombardy also had a more pronounced pattern of two dl-PCB congeners, corresponding to PCB 105 and PCB 118 (Figure 4B). In addition to particular PCB-containing technical formulations used in the past, pollution emissions from the steel sector, cement furnaces, and incineration facilities have been linked to the prevalence of these chemical species in food and feed [62]. Northern Italy hosted chemical industries producing chlorine compounds and actually houses a huge number of industrial facilities potentially responsible for the emission of different environmental pollutants [63]. Therefore, as already reported, the risk of contamination of local feed and food with PCDD/Fs and PCBs may be particularly high [37,57,64].

Differences in ndl-PCB patterns among the two regions were particularly evident for three congeners, namely PCB 138, PCB 153, and PCB 180 (Figure 4C). Regardless of the sampling areas, these chemical species were the most abundant among the six ndl-PCB indicators, possibly because of their greater chlorination degree and longer persistence in the tissues of food-producing animals [55].

### 3.2. Dietary Exposure to Dioxins and PCBs through Milk Consumption

#### 3.2.1. Screening-level Assessment via Deterministic Calculations

The UB exposure levels to PCDD/Fs + dl-PCBs due to the consumption of whole, semi-skimmed, and skimmed cow’s milk in all Italian age groups are presented in Table 3. The EWIs ranged from 0.02 to 15.23 pg TEQ/kg bw/week, with the lowest values calculated for mean elderly consumers ingesting skimmed milk and the highest values calculated for high infant consumers (95th percentile) ingesting whole milk. Globally, the EWIs of dioxins and PCBs were found to be higher across all population age groups due to the consumption of whole milk, while, regardless of the type of milk consumed, infants, toddlers, and children were found to be the most exposed population groups (Table 3).

A more thorough investigation revealed that for adolescents, adults, and the elderly, the EWIs from whole and semi-skimmed milk consumption were roughly the same (due to a compensation effect between the higher ingestions of semi-skimmed milk and the higher fat content of whole milk). On the other hand, despite the intermediate dietary consumption rates of skimmed milk, the EWIs associated with the consumption of this product were an order of magnitude lower (Table 3).

The youngest segment of the European population was found to be much more likely to be exposed to dioxins and PCBs than the other groups by other authors. For example, Austrian children were reported to ingest 0.77 pg TEQ/kg bw/day of dioxins and PCBs through the whole diet, with milk and dairy contributing to 65% of the overall intake (EWI of 3.50 pg TEQ/kg bw/week) [65]. For French children, EWIs of 0.11–0.63 and 0.21–0.69 (0.77–4.41 and 1.47–4.83 pg TEQ/kg bw/week) were reported in relation to the consumption of cow milk and milk plus dairy products, respectively [66].

**Figure 5:**
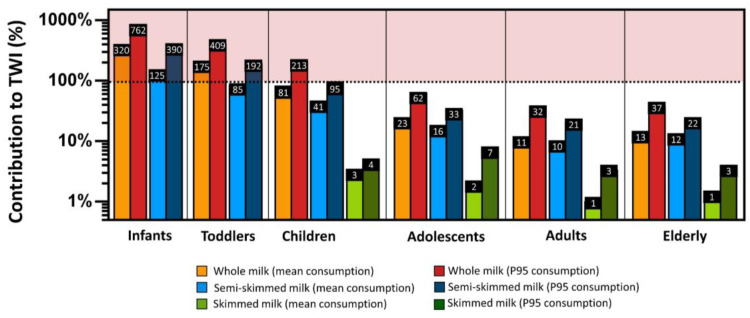
Mean and 95th percentile contribution percentages of whole, semi-skimmed, and skimmed milk consumption by different Italian population age groups to the TWI of PCDD/Fs + dl-PCBs calculated via deterministic methods.

As for Italian children, the whole diet was estimated to be responsible for a weekly intake of 1.98–4.98 pg TEQ/kg bw/day, with milk alone contributing to 10% (EWI of 1.38–3.48 pg TEQ/kg bw/week) and, when combined with dairy products, to 34% (EWI of 4.71–11.85 pg TEQ/kg bw/week) of the weekly ingestions of these contaminants [67]. Similarly, in another study, 1.50–2.64 pg/TEQ/kg bw/week of dioxins and PCBs were estimated to be taken by Italian children through the consumption of milk and dairy products [56]. Hence, based on the calculated EWIs and the conventional deterministic risk characterization analysis summarized in Figure 5, a potential health risk concern related to the consumption of whole and semi-skimmed milk emerged, with infants, toddlers, and children being the populations at higher risk. Indeed, average intakes significantly exceeded the toxicological reference values, being 125–320% (95th percentile: 390–762%) and 85–175% (95th percentile: 192–409%) of the TWI for infants and toddlers, respectively (Figure 5). As for children, the contribution of whole and semi-skimmed milk consumption to the TWI was lower (41–82%; 95th percentile: 95–213%) but still concerning, given that many foods, besides milk, provide significant amounts of dioxins and PCBs (Figure 5).

Our risk characterization provides more alarming results compared to those previously reported in Italy, according to which cow milk contributed roughly 20–30% of the TWI [37,53]. Nevertheless, it should be noted that a direct comparison can be misleading since these studies used old dioxin TEF values or compared the exposure level to the Tolerable Daily Intake (TDI) of 2 pg WHO-TEQ/kg bw, which is seven times lower than the actual TWI. Additionally, the conservative nature and simplifications of the deterministic exposure assessment, which means lacking information about the variability and uncertainty in both food consumption statistics and contamination values, typically lead to incomplete or inaccurate (i.e., overestimated) exposure levels, giving information on the possibility but not the probability of risk.

#### 3.2.2. Refined Assessment via Probabilistic Modelling

Monte Carlo simulations were run to estimate in a more accurate and realistic way the distribution of EWIs and the contribution of milk consumption to the TWI and, therefore, to quantify the probability of the health risk occurring. Only those exposure scenarios of particular concern resulting from the deterministic assessment were simulated. Moreover, since the imputation method for the treatment of data below the LOQ might have a large effect on the forecasted outcomes, simulations were run three separate times using UB, MB, or LB concentrations one at a time.

**Figure 6:**
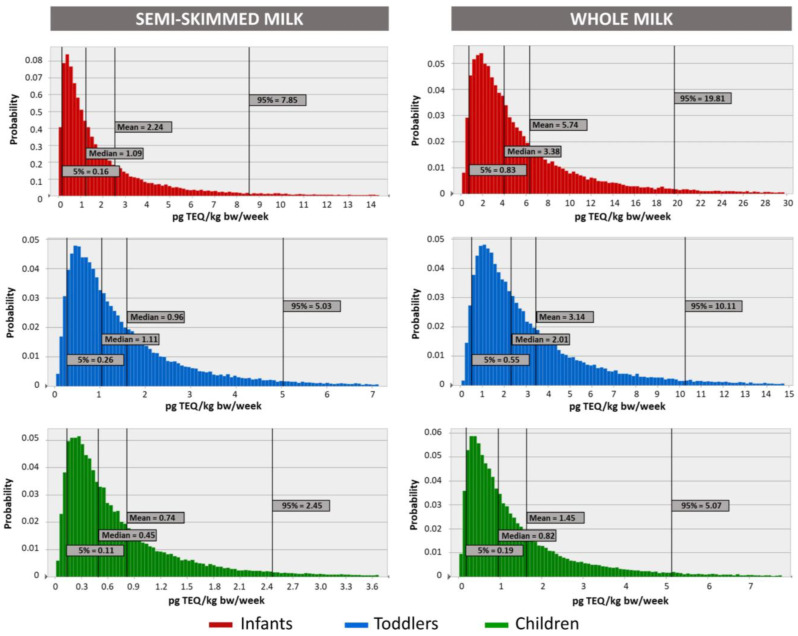
Probability distribution functions calculated via probabilistic Monte Carlo simulations of EWIs of PCDD/Fs + dl-PCBs for infants, toddlers, and children consuming semi-skimmed and whole milk.

Figure 6 presents the variability in the form of distribution frequencies of EWIs at the MB scenario for the most vulnerable population consuming semi-skimmed and whole milk. Average EWIs were estimated to range from 5.74 ± 7.77 pg/TEQ/kg bw/week for infants consuming whole milk to 0.74 ± 0.97 pg/TEQ/kg bw/week in children consuming semi-skewed milk. The median (P50) MB values were significantly lower when compared to the mean values (Figure 6) because of the right-skewness of the distribution frequency curve, which resulted as such because of the right-skewness of both consumption data and contamination levels used as input values for simulations. This condition led to greater robustness of EWIs at the left tail of the curve (lower percentiles). On the contrary, a greater degree of uncertainty is associated with the interpretation of EWIs beyond the 90th percentile since they are not supported by an adequate number of observations. In addition, from the comparison of EWIs at different 10% increment percentiles (Table 4), it was observed that there was a significant amount of uncertainty due to the method for the imputation of data below the LOQ. Indeed, the MB estimates were roughly 15% higher or lower than the LB or UB estimates, respectively. All these findings suggest a possible overestimation of the higher percentile intakes.

Similar considerations also apply to the outcomes resulting from the Monte Carlo-simulated risk characterization associated with chronic exposure to dioxins and PCBs. The MB 95th percentile contribution percentages of milk ingestion to the TWI ranged, on average, from a minimum of 22% for children ingesting semi-skimmed milk to a maximum of 169% for infants ingesting whole milk (Table 5), being significantly lower than those resulting from the deterministically calculated worst case scenario (Figure 5). Nevertheless, as it can be observed from Table 5, the consumption of whole milk by infants was already above the TWI at the MB 40th percentile, indicating that this population group is at particular risk due to the intake of dioxins and PCBs. At the 80th percentile, only the consumption of semi-skimmed milk by children was below the TWI.

**Figure 7:**
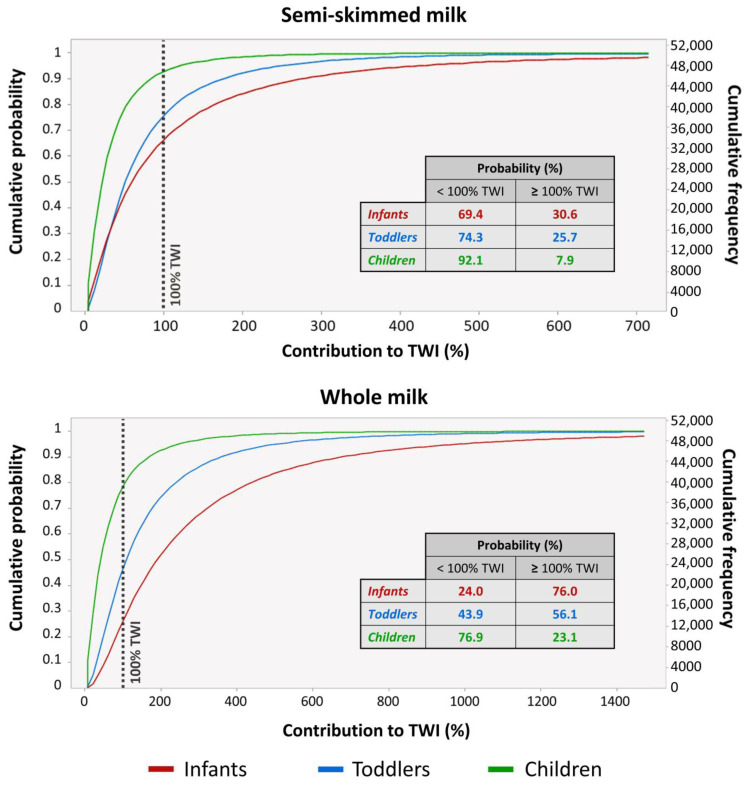
Cumulative probability distribution chart of risk (contribution percentage to the TWI) for infants, toddlers, and children consuming semi-skimmed and whole milk resulting from probabilistic Monte Carlo simulations.

To quantify the uncertainty around when each exposure scenario can occur, the cumulative probability distributions of milk intake percentage contributions to the TWI for infants, toddlers, and children were plotted in Figure 7. Ultimately, the probability for infants to exceed the TWI was 76% for the ingestion of whole milk and 35% for the ingestion of semi-skimmed milk, which means that three out of four and one out of three infants are at a very high risk of chronic toxicity. Moreover, 56% and 26% of toddlers, as well as 22% and 8% of children consuming whole and semi-skimmed milk, respectively, can be exposed to dioxin and PCB levels above the toxicological reference values (Figure 7). Such high percentages were already reported for infants and toddlers through the whole diet [68], but our findings might have more severe implications from a health perspective, given that they are linked to the sole consumption of cow milk, while the diet includes many other sources of these contaminants such as meat, fish, and eggs [3].

Despite these results and the high confidence in probabilistic predictions, there are a few limitations related to the applied methodology that need to be mentioned. In particular, regardless of the substitution method applied, the precision of the measured contaminants may have been biased by the left censorship of these data. Dietary intakes of milk by the general Italian population may not accurately reflect those of the inhabitants of northern Italy due to potential regional variations in dietary habits. Moreover, it was assumed that the entire amount of cow milk consumed originated from local farms in Lombardy and Emilia-Romagna, while commercial products can usually be a mixture of milk from different European (or non-European) countries. The unavailability of data concerning the distribution of the population’s body weight might have been another important limiting factor, which, during probabilistic calculations, has not made it possible to evaluate its impact on the output of the exposure assessment. Finally, the potential variation in the bioavailability of dioxins and PCBs due to milk processing and/or consumer age was not taken into consideration.

## 4. Conclusions

Despite confirming a significant downward trend in dioxin and PCB concentrations within European food and feed chains, this study raises important questions about the potential health risks associated with dietary long-term exposure to these contaminants for the youngest population. By applying probabilistic techniques, a more accurate and realistic picture of exposure levels to dioxins and PCBs and associated risks were achieved, which allowed estimating that the sole consumption of whole cow milk (albeit broadly compliant with maximum EU limits) may lead to exceeding the tolerable weekly intake in approximately 76, 56, and 22% of infants, toddlers, and children, respectively. This output, which is mainly the consequence of the recent seven-fold reduction in the tolerable weekly intake of dioxins and PCB, clearly worsens the overall risk profile of the young population and underscores the need for a multi-pronged approach involving regulatory measures and public education to reduce dietary exposure further. In this setting, monitoring and surveillance systems represent an essential tool to identify contamination sources and track human exposure. On the other hand, effective communication of both risks and benefits associated with the consumption of milk by vulnerable consumers is necessary. This may include promoting a varied diet and alternating with infant formulae, follow-up formulae, and other baby foods for which stricter limits of dioxins and PCBs are in force.

## Figures and Tables

**Table 1:** Summary of PCDD/F, dl-PCB, and ndl-PCB mean concentrations in cow milk samples collected in Lombardy and Emilia-Romagna regions (Italy) over the 2018–2021 sampling years. Concentrations of PCDD/Fs, dl-PCBs, and PCDD/Fs + dl-PCBs are reported both in pg/g fat and in pg TEQ/g fat (in square brackets). Concentrations of ndl-PCBs are reported in ng/g fat.

	∑PCDD/Fs			∑dl-PCBs			∑PCDD/Fs + dl-PCBs			∑ndl-PCBs		
	Mean	SD	Median	Mean	SD	Median	Mean	SD	Median	Mean	SD	Median
Region												
Lombardy	2.07 [0.23]	0.5 [0.06]	1.92 [0.21]	764 [0.66]	1238 [0.61]	546 [0.53]	766 [0.88]	1238 [0.63]	547 [0.75]	6.71	2.65	6.00
Emilia-Romagna	1.77 [0.19]	0.39 [0.04]	1.63 [0.18]	740 [0.41]	534.54 [0.28]	597.94 [0.34]	741.76 [0.60]	534.55 [0.30]	599.71 [0.52]	6.25	1.07	6.00
Year												
2018	1.9 [0.22]	0.39 [0.06]	1.78 [0.20]	1015 [0.59]	1258 [0.47]	706 [0.46]	1017 [0.81]	1258 [0.50]	707 [0.69]	6.66	1.70	6.00
2019	1.94 [0.21]	0.47 [0.05]	1.82 [0.20]	1079 [0.63]	995 [0.74]	837 [0.49]	1081 [0.82]	995 [0.77]	838 [0.70]	6.79	3.60	6.00
2020	2.07 [0.23]	0.48 [0.07]	1.95 [0.20]	883 [0.56]	489 [0.39]	700 [0.44]	885 [0.79]	490 [0.42]	703 [0.69]	6.28	0.82	6.00
2021	2.04 [0.20]	0.44 [0.03]	1.96 [0.19]	783 [0.43]	437 [0.25]	628 [0.35]	785 [0.63]	437 [0.27]	630 [0.55]	6.12	0.32	6.00

**Table 2:** Comparison of PCDD/F, dl-PCB, and ndl-PCB mean concentrations in cow milk samples collected in Lombardy and Emilia-Romagna regions (Italy) over the 2018–2021 sampling years with the previous 2012–2014 survey [37]. Concentrations of PCDD/Fs, dl-PCBs, and PCDD/Fs + dl-PCBs are reported both in pg/g fat and in pg TEQ/g fat (in square brackets). Concentrations of ndl-PCBs are reported in ng/g fat.

Sampling Years	2012–2014		2018–2021		Variation (%)	
Region	Lombardy	Emilia-Romagna	Lombardy	Emilia-Romagna	Lombardy	Emilia-Romagna
∑PCDD/Fs	3.21 [0.35]	3.31 [0.34]	2.07 [0.23]	1.77 [0.19]	−36 [−34]	−47 [−44]
∑DL-PCBs	2513 [1.14]	1591 [0.65]	764 [0.66]	740 [0.41]	−70 [−42]	−53 [−37]
∑PCDD/Fs + dl-PCBs	2517 [1.49]	1595 [0.98]	766 [0.88]	742 [0.60]	−70 [−41]	−53 [−39]
∑6 ndl-PCBs	10.59	7.73	6.71	6.25	−37	−19

**Table 3:** Estimated weekly intake (EWI, pg TEQ/kg bw/week) to PCDD/Fs + dl-PCBs of different Italian population age groups due to the chronic mean and high (95th percentile, in square brackets) consumption of whole (3.5 g fat/100 g), semi-skimmed (1.8 g fat/100 g) and skimmed (0.3 g fat/100 g) cow milk containing average concentrations of 0.78 pg TEQ/g fat of PCDD/Fs + dl-PCBs.

Population Group	Type of Milk	Weekly Consumption	EWI
		(g/kg bw/week)	(pg TEQ/kg bw week)
Infants	Semi-skimmed	178.5 [555.87]	2.51 [7.80]
	Whole	234.15 [557.89]	6.39 [15.23]
Toddlers	Semi-skimmed	120.68 [272.86]	1.69 [3.83]
	Whole	128.1 [299.32]	3.50 [8.17]
Children	Skimmed	53.76 [141.19]	0.06 [0.09]
	Semi-skimmed	58.08 [135.1]	0.83 [1.90]
	Whole	59.08 [156.1]	1.61 [4.26]
Adolescents	Skimmed	20.65 [58.66]	0.05 [0.14]
	Semi-skimmed	22.75 [47.39]	0.32 [0.67]
	Whole	17.08 [45.71]	0.47 [1.25]
Adults	Skimmed	11.13 [25.41]	0.03 [0.06]
	Semi-skimmed	14.42 [29.33]	0.20 [0.41]
	Whole	8.12 [23.45]	0.22 [0.64]
Elderly	Skimmed	10.50 [25.41]	0.02 [0.06]
	Semi-skimmed	16.87 [30.66]	0.24 [0.43]
	Whole	9.24 [27.23]	0.25 [0.74]

**Table 4:** Percentiles from Monte Carlo simulations of estimated weekly intake (EWI, pg TEQ/kg bw/week) of PCDD/Fs + dl-PCBs through milk consumption.

Population Group	Simulation Scenario	Milk Type	Percentiles								
			10th	20th	30th	40th	50th	60th	70th	80th	90th
Infants	LB	Semi-skimmed	0.17	0.3	0.45	0.64	0.88	1.22	1.73	2.58	4.48
		Whole	0.68	1.1	1.56	2.1	2.78	3.65	4.92	6.97	11.25
	MB	Semi-skimmed	0.22	0.38	0.57	0.79	1.07	1.46	2.03	2.97	5.08
		Whole	0.9	1.42	1.97	2.59	3.38	4.38	5.76	7.98	12.63
	UB	Semi-skimmed	0.28	0.46	0.67	0.92	1.24	1.68	2.31	3.35	5.66
		Whole	1.17	1.77	2.4	3.11	3.96	5.05	6.52	8.88	13.71
Toddlers	LB	Semi-skimmed	0.35	0.52	0.69	0.88	1.11	1.39	1.78	2.39	3.61
		Whole	0.74	1.08	1.44	1.84	2.33	2.89	3.68	4.92	7.39
	MB	Semi-skimmed	0.28	0.42	0.58	0.75	0.96	1.22	1.59	2.16	3.31
		Whole	0.58	0.88	1.21	1.56	1.99	2.53	3.28	4.44	6.75
	UB	Semi-skimmed	0.36	0.53	0.72	0.88	1.11	1.39	1.78	2.38	3.59
		Whole	0.76	1.11	1.46	1.86	2.32	2.9	3.69	4.92	7.3
Children	LB	Semi-skimmed	0.09	0.15	0.21	0.27	0.36	0.47	0.63	0.88	1.42
		Whole	0.27	0.41	0.57	0.74	0.96	1.24	1.63	2.26	3.55
	MB	Semi-skimmed	0.12	0.19	0.26	0.35	0.45	0.58	0.75	1.03	1.61
		Whole	0.21	0.34	0.47	0.63	0.82	1.08	1.44	2.01	3.24
	UB	Semi-skimmed	0.27	0.42	0.57	0.75	0.97	1.24	1.64	2.25	3.51
		Whole	0.28	0.46	0.67	0.92	1.24	1.68	2.31	3.35	5.66

**Table 5:** Percentiles from Monte Carlo simulations of the risk characterization of PCDD/Fs + dl-PCBs expressed as percentage contribution to the Tolerable weekly intake (% TWI).

Population Group	Simulation Scenario	Milk Type	Percentiles								
			10th	20th	30th	40th	50th	60th	70th	80th	90th
Infants	LB	Semi-skimmed	8.54	15.05	22.62	31.81	44.11	60.91	86.47	128.79	224.04
		Whole	33.83	54.98	77.88	104.98	138.96	182.39	246.23	348.6	562.56
	MB	Semi-skimmed	11.23	19.16	28.36	39.42	53.68	73.14	101.52	148.56	254.19
		Whole	45.15	71.16	98.31	129.71	169	218.78	287.98	398.29	631.44
	UB	Semi-skimmed	13.75	23.15	33.57	46.24	61.97	83.81	115.72	167.39	283.14
		Whole	58.71	88.56	119.85	155.49	197.89	252.29	326.13	443.77	685.62
Toddlers	LB	Semi-skimmed	17.71	25.96	34.43	43.93	55.26	69.42	89.06	119.5	180.31
		Whole	37.09	54.25	72.02	91.85	115	144.59	184.04	245.99	369.72
	MB	Semi-skimmed	13.95	21.21	28.86	37.46	47.89	61.18	79.51	108.23	165.22
		Whole	28.94	44.24	59.91	77.97	99.42	126.37	164.16	221.88	337.53
	UB	Semi-skimmed	17.92	26.36	34.81	44.2	55.46	69.71	89.12	119.23	179.64
		Whole	38.09	55.67	73.03	92.82	116.04	144.94	184.53	244.97	364.84
Children	LB	Semi-skimmed	4.61	7.36	10.28	13.74	18.03	23.62	31.59	44.14	70.96
		Whole	13.42	20.63	28.25	37.16	47.99	61.91	81.47	112.98	177.36
	MB	Semi-skimmed	6.08	9.59	13.14	17.26	22.28	28.79	37.57	51.74	80.64
		Whole	10.59	16.91	23.73	31.49	41.16	53.99	71.85	100.38	160.07
	UB	Semi-skimmed	7.96	11.89	15.91	20.44	25.88	32.86	42.40	57.38	87.00
		Whole	13.73	21.09	28.8	37.65	48.53	62.57	82.11	112.62	176.55

## Data Availability

The dataset generated during the current study will be made available upon reasonable request.

## Supplementary Materials

The following supporting information can be downloaded at: https://www.mdpi.com/article/10.3390/foods12091869/s1, Table S1: Limit of quantification (LOQ) and recovery values of the 35 measured PCDD/F and PCB congeners. Table S2: Mean concentrations of the 35 measured PCDD/F and PCB congeners in milk samples from the two Italian regions. Table S3: Mean concentrations of the 35 measured PCDD/F and PCB congeners in milk samples collected during the 4-year sampling plan.
