# Supplementary material for: Safety Evaluation and Probabilistic Health Risk Assessment of Cow Milk Produced in Northern Italy According to Dioxins and PCBs Contamination Levels
Source: Foods. 2023 Apr 30;12(9):1869. doi: 10.3390/foods12091869 (PMC10178383; doi:10.3390/foods12091869)
Supplement: Supplementary file 1 [file foods-12-01869-s001.zip › foods-2353776-supplementary.pdf]

Article

Safety Evaluation and Probabilistic Health Risk Assessment of Cow Milk produced in Northern Italy according to Dioxins and PCBs Contamination Levels

Maria Olga Varrà [1], Valentina Lorenzi [2, *], Emanuela Zanardi [1], Simonetta Menotta [2], Giorgio Fedrizzi [3], Barbara Angelone [2], Mara Gasparini [2], Francesca Fusi [2], Stefano Foschini [4], Anna Padovani [5] and Sergio Ghidini [1]

[1] Department of Food and Drug, University of Parma, Parma, Italy; mariaolga.varra@unipr.it; emanuela.zanardi@unipr.it; sergio.ghidini@unipr.it.
[2] Istituto Zooprofilattico Sperimentale della Lombardia e dell'Emilia Romagna "Bruno Ubertini", Brescia, Italy; valentina.lorenzi@izsler.it; simonetta.menotta@izsler.it; barbara.angelone@izsler.it; mara.gasparini@izsler.it; francesca.fusi@izsler.it
[3] Chemical Department, Istituto Zooprofilattico Sperimentale della Lombardia e dell'Emilia-Romagna "Bruno Ubertini", Bologna, Italy; giorgio.fedrizzi@izsler.it.
[4] Unità Organizzativa Veterinaria, Direzione Generale Welfare Regione Lombardia, Milano, Italy; stefano.foschini@regione.lombardia.it.
[5] Area Sanità Veterinaria e Igiene degli Alimenti, Settore Prevenzione Collettiva e Sanità Pubblica, Direzione Generale Cura della Persona, Salute e Welfare, Regione Emilia Romagna, Bologna, Italy; anna.padovani@regione.emilia-romagna.it
* Correspondence: valentina.lorenzi@izsler.it; Tel.: +390302290799

SUPPLEMENTARY MATERIALS

Table S1. Limit of quantification (LOQ) and recovery values of the 35 measured PCDD/F and PCB congeners.

Congener	LOQ	Average percent recovery (%)[1]	Standard deviation of percent recovery (%)[1]	Internal standard recovery range (%)	
				Min	Max
**2,3,7,8-TCDF** (2,3,7,8-Tetrachlorodibenzofuran)	0.04 pg/g fat	105	16	45	169
**1,2,3,7,8-PeCDF** (1,2,3,7,8-Pentachlorodibenzofuran)	0.04 pg/g fat	96	13	45	185
**2,3,4,7,8-PeCDF** (2,3,4,7,8-Pentachlorodibenzofuran)	0.04 pg/g fat	100	14	45	178
**1,2,3,4,7,8-HxCDF** (1,2,3,4,7,8 Hexachlorodibenzofuran)	0.1 pg/g fat	100	15	45	152
**1,2,3,6,7,8-HxCDF** (1,2,3,6,7,8-Hexachlorodibenzofuran)	0.1 pg/g fat	98	13	45	123
**2,3,4,6,7,8-HxCDF** (2,3,4,6,7,8-Hexachlorodibenzofuran)	0.1 pg/g fat	101	17	45	136
**1,2,3,7,8,9-HxCDF** (1,2,3,7,8,9-Hexachlorodibenzofuran)	0.1 pg/g fat	99	15	45	147
**1,2,3,4,6,7,8-HpCDF** (1,2,3,4,6,7,8-Heptachlorodibenzofuran)	0.1 pg/g fat	99	14	45	143
**1,2,3,4,7,8,9-HpCDF** (1,2,3,4,7,8,9-Heptachlorodibenzofuran)	0.1 pg/g fat	99	19	45	138
**1,2,3,4,6,7,8,9-OCDF** (1,2,3,4,6,7,8,9-Octachlorodibenzofuran)	0.2 pg/g fat	107	21	N/A[2]	N/A[2]
**2,3,7,8-TCDD** (2,3,7,8-Tetrachlorodibenzodioxin)	0.04 pg/g fat	105	15	45	164
**1,2,3,7,8-PeCDD** (1,2,3,7,8-Pentachlorodibenzodioxin)	0.04 pg/g fat	104	15	45	181
**1,2,3,4,7,8-HxCDD** (1,2,3,4,7,8-Hexachlorodibenzodioxin)	0.1 pg/g fat	101	14	45	141
**1,2,3,6,7,8-HxCDD** (1,2,3,6,7,8-Hexachlorodibenzodioxin)	0.1 pg/g fat	100	15	45	130
**1,2,3,7,8,9-HxCDD** (1,2,3,7,8,9-Hexachlorodibenzodioxin)	0.1 pg/g fat	105	19	N/A[3]	N/A[3]
**1,2,3,4,6,7,8-HpCDD** (1,2,3,4,6,7,8-Heptachlorodibenzodioxin)	0.1 pg/g fat	102	14	45	140
**1,2,3,4,6,7,8,9-OCDD** (1,2,3,4,6,7,8,9-Octachlorodibenzodioxin)	0.2 pg/g fat	106	14	45	157
**PCB 81** (3,4,4',5-Tetrachlorobiphenyl)	10 pg/g fat	100	13	45	145
**PCB 77** (3,3',4,4'-Tetrachlorobiphenyl)	10 pg/g fat	94	11	45	145
**PCB 123** (2',3,4,4',5-Pentachlorobiphenyl)	10 pg/g fat	101	12	45	145
**PCB 118** (2,3',4,4',5-Pentachlorobiphenyl)	100 pg/g fat	99	15	45	145
**PCB 114** (2,3,4,4',5-Pentachlorobiphenyl)	10 pg/g fat	101	11	45	145
**PCB 105** (2,3,3',4,4'-Pentachlorobiphenyl)	100 pg/g fat	92	12	45	145

Table S1. *Cont.*

Congener	LOQ	Average percent recovery (%)[1]	Standard deviation of percent recovery (%)[1]	Internal standard recovery range %	
				Min	Max
**PCB 126** (3,3',4,4',5-Pentachlorobiphenyl)	1 pg/g fat	107	13	45	145
**PCB 167** (2,3',4,4',5,5'-Hexachlorobiphenyl)	10 pg/g fat	106	10	45	145
**PCB 156** (2,3,3',4,4',5-Hexachlorobiphenyl)	100 pg/g fat	101	13	45	145
**PCB 157** (2,3,3',4,4',5'-Hexachlorobiphenyl)	10 pg/g fat	99	11	45	145
**PCB 169** (3,3',4,4',5,5'-Hexachlorobiphenyl)	1 pg/g fat	101	12	45	145
**PCB 189** (2,3,3',4,4',5,5'-Heptachlorobiphenyl)	10 pg/g fat	106	14	45	145
**PCB 28** (2,4,4'-Trichlorobiphenyl)	1 ng/g fat	102	15	45	145
**PCB 52** (2,2',5,5'-Tetrachlorobiphenyl)	1 ng/g fat	105	12	N/A[3]	N/A[3]
**PCB 101** (2,2',4,5,5'-Pentachlorobiphenyl)	1 ng/g fat	104	12	N/A[3]	N/A[3]
**PCB 153** (2,2',4,4',5,5'-Hexachlorobiphenyl)	1 ng/g fat	108	15	N/A[2]	N/A[2]
**PCB 138** (2,2',3,4,4',5'-Hexachlorobiphenyl)	1 ng/g fat	109	13	N/A[3]	N/A[3]
**PCB 180** (2,2',3,4,4',5,5'-Heptachlorobiphenyl)	1 ng/g fat	102	14	45	145

[1] As reported in the "United States Environmental Protection Agency Office of Water Engineering and Analysis Division US EPA Method 1613/B. Tetra-through octa-chlorinated dioxins and furans by isotope dilution HRGC/HRMS: Method 1613 Revision B October 1994" and in the "United States Environmental Protection Agency Office of Water Engineering and Analysis Division US EPA Method 1668/C. Chlorinated biphenyl congeners in water, soil, sedi- ment, biosolids, and tissue by HRGC/HRMS: Method 1668 Revision C April 2010".

[2] N/A: Not available, the congener is not present in the used internal standards.

[3] N/A: Not available, the congener is present in the injection solution added at the end of the process.

Table S2: Mean concentrations of the 35 measured PCDD/F and PCB congeners in milk samples from the two Italian regions.

	Sampling Region			
**PCDD/Fs**	**Emilia-Romagna**		**Lombardy**	
	(pg/g fat)	(pg TEQ/g fat)	(pg/g fat)	(pg TEQ/g fat)
**2,3,7,8-TCDD**	0.040	0.002	0.042	0.002
**2,3,7,8-TCDF**	0.051	0.003	0.069	0.003
**1,2,3,7,8-PeCDD**	0.040	0.013	0.050	0.013
**1,2,3,7,8-PeCDF**	0.042	0.000	0.043	0.000
**2,3,4,7,8-PeCDF**	0.089	0.035	0.152	0.036
**1,2,3,4,7,8-HxCDD**	0.101	0.000	0.100	0.000
**1,2,3,4,7,8-HxCDF**	0.107	0.006	0.132	0.007
**1,2,3,6,7,8-HxCDD**	0.102	0.002	0.106	0.002
**1,2,3,6,7,8-HxCDF**	0.101	0.002	0.109	0.002
**1,2,3,7,8,9-HxCDD**	0.100	0.001	0.103	0.001
**1,2,3,7,8,9-HxCDF**	0.101	0.000	0.100	0.000
**2,3,4,6,7,8-HxCDF**	0.105	0.003	0.112	0.003
**1,2,3,4,6,7,8-HpCDD**	0.138	0.000	0.179	0.000
**1,2,3,4,6,7,8-HpCDF**	0.127	0.000	0.119	0.000
**1,2,3,4,7,8,9-HpCDF**	0.101	0.000	0.100	0.000
**1,2,3,4,6,7,8,9-OCDD**	0.232	0.000	0.336	0.000
**1,2,3,4,6,7,8,9-OCDF**	0.189	0.000	0.216	0.000
**dl-PCBs**				
**PCB 77**	11.524	0.000	10.284	0.000
**PCB 81**	10.297	0.000	10.000	0.000
**PCB 105**	127.665	0.004	190.428	0.004
**PCB 114**	12.705	0.000	16.594	0.000
**PCB 118**	417.261	0.017	715.452	0.018
**PCB 123**	11.978	0.000	14.009	0.000
**PCB 126**	3.451	0.484	5.983	0.501
**PCB 156**	101.861	0.001	119.034	0.001
**PCB 157**	11.513	0.000	20.312	0.000
**PCB 167**	20.283	0.000	42.682	0.000
**PCB 169**	1.293	0.008	1.108	0.009
**PCB 189**	10.165	0.001	12.129	0.001

**ndl-PCBs**	**Emilia-Romagna**	**Lombardy**
	(ng/g fat)	(ng/g fat)
**PCB 28**	1.046	1.000
**PCB 52**	1.003	1.000
**PCB 101**	1.005	1.000
**PCB 138**	1.114	1.240
**PCB 153**	1.082	1.395
**PCB 180**	1.004	1.071

Table S3. Mean concentrations of the 35 measured PCDD/F and PCB congeners in milk samples collected during the 4-year sampling plan.

	Sampling Year							
	2018		2019		2020		2021	
PCDD/Fs	(pg/g fat)	(pg TEQ/g fat)	(pg/g fat)	(pg TEQ/g fat)	(pg/g fat)	(pg TEQ/g fat)	(pg/g fat)	(pg TEQ/g fat)
**2,3,7,8-TCDD**	0.043	0.004	0.040	0.001	0.041	0.004	0.040	0.000
**2,3,7,8-TCDF**	0.078	0.006	0.060	0.003	0.048	0.001	0.044	0.001
**1,2,3,7,8-PeCDD**	0.046	0.013	0.049	0.014	0.049	0.019	0.043	0.006
**1,2,3,7,8-PeCDF**	0.043	0.000	0.042	0.000	0.044	0.000	0.040	0.000
**2,3,4,7,8-PeCDF**	0.139	0.038	0.122	0.033	0.153	0.045	0.103	0.027
**1,2,3,4,7,8-HxCDD**	0.100	0.000	0.100	0.000	0.100	0.000	0.102	0.001
**1,2,3,4,7,8-HxCDF**	0.125	0.006	0.124	0.009	0.128	0.007	0.115	0.003
**1,2,3,6,7,8-HxCDD**	0.103	0.001	0.102	0.001	0.109	0.002	0.110	0.003
**1,2,3,6,7,8-HxCDF**	0.106	0.002	0.105	0.002	0.110	0.003	0.105	0.002
**1,2,3,7,8,9-HxCDD**	0.102	0.001	0.101	0.000	0.104	0.001	0.101	0.000
**1,2,3,7,8,9-HxCDF**	0.100	0.000	0.101	0.000	0.101	0.000	0.100	0.000
**2,3,4,6,7,8-HxCDF**	0.105	0.002	0.106	0.002	0.122	0.005	0.113	0.003
**1,2,3,4,6,7,8-HpCDD**	0.153	0.000	0.157	0.000	0.194	0.001	0.181	0.000
**1,2,3,4,6,7,8-HpCDF**	0.110	0.000	0.106	0.000	0.128	0.000	0.167	0.000
**1,2,3,4,7,8,9-HpCDF**	0.100	0.000	0.100	0.000	0.102	0.000	0.100	0.000
**1,2,3,4,6,7,8,9-OCDD**	0.253	0.000	0.307	0.000	0.341	0.000	0.359	0.000
**1,2,3,4,6,7,8,9-OCDF**	0.193	0.000	0.220	0.000	0.202	0.000	0.222	0.000
**dl-PCBs**								
PCB 77	10.725	0.000	11.465	0.000	10.000	0.000	10.043	0.000
PCB 81	10.265	0.000	10.000	0.000	10.000	0.000	10.000	0.000
PCB 105	189.480	0.004	174.719	0.004	144.357	0.009	132.560	0.002
PCB 114	16.654	0.000	15.922	0.000	13.930	0.000	12.006	0.000
PCB 118	689.487	0.021	652.492	0.019	0.016	0.016	448.680	0.013
PCB 123	14.221	0.000	13.813	0.000	12.447	0.000	11.074	0.000
PCB 126	5.159	0.506	5.665	0.562	5.008	0.494	4.093	0.364
PCB 156	114.159	0.001	120.687	0.001	106.707	0.000	103.331	0.000
PCB 157	18.336	0.000	18.102	0.000	16.303	0.000	14.202	0.000
PCB 167	34.110	0.000	42.618	0.000	32.942	0.000	25.228	0.000
PCB 169	1.312	0.013	1.004	0.001	1.039	0.003	1.266	0.015
PCB 189	11.624	0.000	12.168	0.000	11.179	0.007	10.027	0.000

ndl-PCBs	2018 (ng/g fat)	2019 (ng/g fat)	2020 (ng/g fat)	2021 (ng/g fat)
**PCB 28**	1.029	1.016	1.000	1.000
**PCB 52**	1.000	1.003	1.000	1.000
**PCB 101**	1.000	1.006	1.000	1.000
**PCB 138**	1.257	1.281	1.058	1.035
**PCB 153**	1.336	1.378	1.208	1.083
**PCB 180**	1.037	1.108	1.014	1.000
